# Evaluation of the microperimetry in eyes with cuticular drusen

**DOI:** 10.1038/s41598-022-22513-5

**Published:** 2022-10-20

**Authors:** Seung Wan Nam, Jung Hwa Lee, Zeeyoon Byun, Don-Il Ham, Mingui Kong

**Affiliations:** 1 Department of Ophthalmology, Samsung Medical Center, Sungkyunkwan University School of Medicine, Seoul, Republic of Korea; 2 Department of Ophthalmology, HanGil Eye Hospital, 35 Bupyeong-Daero, Bupyeong-Gu, Incheon, 21388 Republic of Korea; 3 Department of Ophthalmology, College of Medicine, Catholic Kwandong University, Incheon, Republic of Korea

**Keywords:** Diseases, Medical research

## Abstract

Retinal sensitivity may vary by subtypes of cuticular drusen. This retrospective study included 52 eyes of 32 patients with cuticular drusen. All the patients underwent assessment of best-corrected visual acuity (BCVA), spectral-domain optical coherence tomography (SD-OCT), color fundus photography, fluorescein angiography, fundus autofluorescence, and microperimetry. The area occupied by drusen was counted using microperimetry. The cuticular drusen subtype was classified into 3 groups based on the SD-OCT findings. Age, BCVA, pattern standard deviation, area occupied by drusen, pupil size, and the false-positive rate were not significantly different (p > 0.05) according to the cuticular drusen type. The mean retinal sensitivity (MRS) (p = 0.063) and mean deviation (MD) (p = 0.098) showed marginally significant differences among the groups. In the subgroup analyses, type 1 and type 3 cuticular drusen showed significant differences in the MD (− 1.8 ± 2.1 vs − 5.1 ± 5.3; p = 0.011) and MRS (25.1 ± 2.2 vs 21.3 ± 5.7; p = 0.016) without differences in age, BCVA, or the area occupied by drusen (p > 0.05). The results indicate that depending on the subtypes of cuticular drusen type, the deterioration of retinal sensitivity is more likely to occur than decreased vision.

## Introduction

Age-related macular degeneration (AMD) is a leading cause of vision loss in elderly patients living in developed countries^1^. AMD is characterized by the formation of drusen, which can vary in size, shape, and location^2^. Drusen are deposits of cellular debris, and the size, extent, and shape of drusen can affect the risk for advanced AMD^3^. It has been recognized that there are diverse phenotypes of drusen in AMD, including soft drusen, cuticular drusen, reticular pseudodrusen (RPD), and pachydrusen^4–7^.

Cuticular drusen shows fundus findings that include multiple, yellow or pale, small, round lesions with symmetric distribution between bilateral eyes, and these are best visualized on fundus fluorescein angiography (FA) as hyperfluorescent drusen with a typical “stars-in-the-sky” appearance^4,8^. Cuticular drusen are located beneath the retinal pigment epithelium (RPE), with RPE elevation on optical coherence tomography (OCT)^4^. Cuticular drusen are known for their unique composition and morphology, with distinct pathogenesis and different prognoses^4^. Cuticular drusen can be divided into 3 patterns depending on the morphologic features using OCT^4^. Type 1 was defined as a shallow elevation of the RPE basal laminar band, with drusen internal contents difficult to discern; type 2 was defined as a drusen of triangular morphologic characteristics resulting in a saw-tooth appearance and hyporeflective internal contents; and type 3 was defined as a broad, mound-shaped elevation of the RPE basal laminar band with hyporeflective internal contents.

Microperimetry is a non-invasive and effective method for the detection of functional changes^9^. Microperimetry can assess fixation and central visual field defects topographically, and it is a useful tool in that the shape of the macula is not always associated with the function of the macula^10^. Previous studies revealed that drusen formation correlates with photoreceptor degeneration, which is related to decreased retinal sensitivity^10,11^. The ellipsoid zone in OCT has a predictive value for retinal sensitivity^9^, and deterioration of retinal sensitivity precedes earlier than deterioration of visual acuity^11^. However, studies on the microperimetry of cuticular drusen are still scarce due to the low prevalence of cuticular drusen^5^. Furthermore, no studies have been conducted on the microperimetry of cuticular drusen based on OCT according to the cuticular drusen type.

This study aimed to investigate retinal sensitivity in eyes with cuticular drusen and the differences in retinal sensitivity depending on the cuticular drusen type.

## Methods

This study was approved by the Institutional Review Board (IRB) of the HanGil Eye Hospital and adhered to the tenets of the Declaration of Helsinki. Given the retrospective design of this study and the use of anonymized data, requirements for informed consent were waived by the IRB of the HanGil Eye Hospital in Korea.

This retrospective cross-sectional study included patients with cuticular drusen only from May 2020 to March 2021 at the HanGil Eye Hospital. The exclusion criteria were advanced AMD including macular neovascularization and geographic atrophy, coexistence of other types of drusen or drusenoid deposits (e.g. soft drusen, reticular pseudodrusen, and pachydrusen) except cuticular drusen, uveitis, diabetic retinopathy, hypertensive retinopathy, epiretinal membrane, retinal detachment, glaucoma, refractive error exceeding ± 6 diopters, history of retinal laser photocoagulation, ocular media opacity, and insufficient ocular examinations.

All patients underwent a complete ophthalmologic examination, including slit-lamp examination and measurement of the best-corrected visual acuity (BCVA), color fundus photography (CFP; TrueColor Confocal slit scanner, Centervue Spa, a company of iCare Finland Oy; Vantaa, Finland), FA (Spectralis HRA + OCT, Heidelberg Engineering, Heidelberg, Germany or Optos California 200DTx, Dunfermline, United Kingdom), SD-OCT (Spectralis HRA + OCT, Heidelberg Engineering, Heidelberg, Germany), fundus autofluorescence (FAF; Optos California 200DTx, Dunfermline, United Kingdom), and microperimeter (Compass fundus perimeter, CMP; Centervue Spa, a company of iCare Finland Oy; Vantaa, Finland). Multimodal imaging was used to assess the lesions. Patient information including age, sex, and parameter of microperimetric parameters was obtained for each eye.

### Classification of drusen or drusenoid deposits

Cuticular drusen are multiple yellow or pale, small, round lesions observed in the CFP with a symmetric distribution pattern between bilateral eyes. In this study, there had to be at least 50 scattered, uniformly sized, small (25–75 μm) hyperfluorescent drusen with a typical “stars-in-the-sky” appearance on FA images in each eye^4,12^. The lesion had to be located beneath the RPE, with RPE elevation on OCT^4,12^. The types of cuticular drusen were classified by horizontal, vertical, and raster scan images of SD-OCT centered on the fovea based on the shape of the majority of cuticular drusen.

Soft drusen were identified based on the presence of round- or ovoid-shaped drusen with poorly defined borders, which can be tightly packed and even confluent in CFP, and confirmed that the deposit was below the RPE layer using OCT^6^. RPD was determined with multimodal imaging as described in the previous study^13^. Pachydrusen was identified based on the criteria by Spaide^6^.

### Multimodal imaging

CFP was performed (60° horizontally and 55° vertically). The protocol of SD-OCT consisted of two B-scans centered on the fovea (horizontal and vertical, 12.0 mm, ART 100) and raster scans (30° × 20°, 6.0 mm, centered at the center of the fovea, 25 horizontal B-scans, ART 9) using enhanced depth imaging protocols. Automatic real-time (ART) mode using an eye-tracker system was activated. Optos California 200DTx could cover 200° horizontally and 170° vertically.

### Microperimetry

Microperimetry was performed using CMP. All the patients underwent mesopic tests. Prior to testing, pupillary dilation was performed using 1.0% tropicamide. The room light was switched off immediately before each examination. The standard 24–2 grid was used in this study^14^. The testing strategy was the Zippy estimation by sequential testing (ZEST)^14^. ZEST is one of the perimetric algorithms with reasonable error and test time^14,15^. Since CMP assesses wide-field (30°) VF, it is suitable for testing retinal sensitivity in cuticular drusen eyes due to its wide distribution on the fundus.

The mean deviation (MD), pattern standard deviation (PSD), pupil size, and false-positive rate were automatically calculated using CMP. The mean retinal sensitivity (MRS), defined as the arithmetic average of the retinal sensitivities at all points in each test, was manually calculated. Microperimetry tests were considered reliable if the false-positive rate was less than 18%^14^. Active compensation for fixation loss was provided by automated, tracking of eye movements by infrared scanning of the retina.

**Figure 1:**
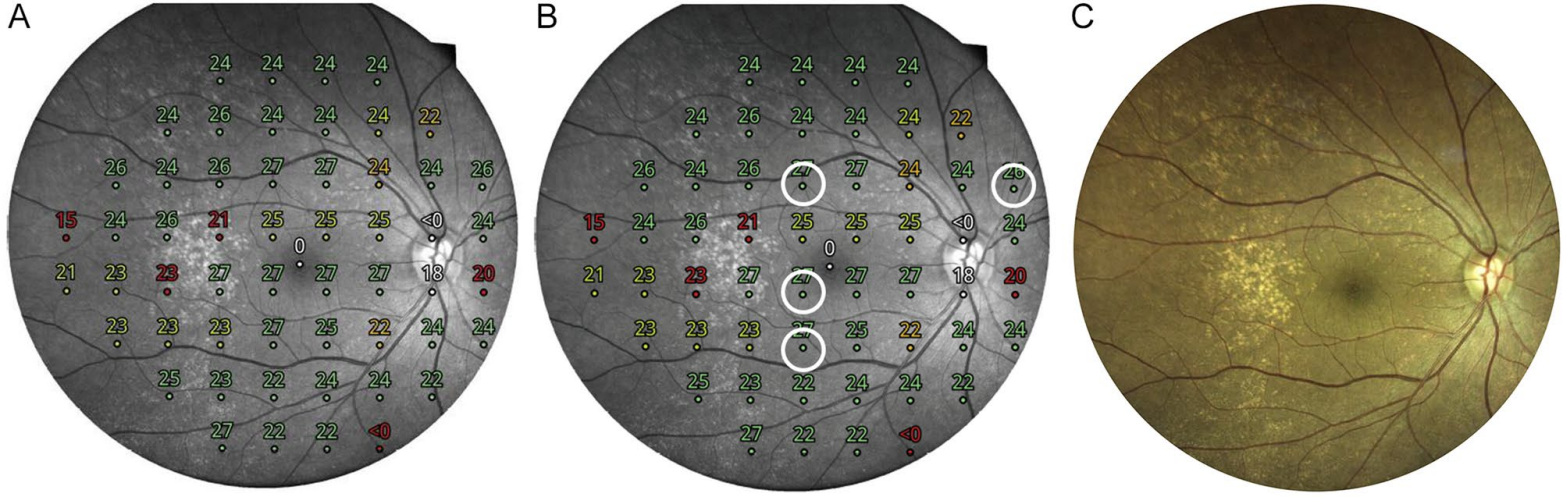
Measurement of retinal sensitivity using microperimetry. Retinal sensitivity (decibels [dB]) for each of the 52 areas was measured using the compass fundus microperimeter. (**A**) Composited images of topographical information on retinal sensitivity and red free fundus photographs were obtained. (**B**) Areas unoccupied by drusen were identified manually in composited images. (**C**) A color fundus photograph was used to confirm the area occupied by drusen.

To determine the extent of cuticular drusen, the area occupied by the drusen was manually determined in a superimposed fundus image automatically generated by CMP. Superimposed fundus images are composite of topographical information on retinal sensitivity and red free fundus photographs. The total number of areas was 52, and if there was even one cuticular drusen in the area, it was considered occupied. A color fundus photograph was used to confirm the area occupied by drusen, manually and we counted the number of areas occupied by drusen (Fig. 1). All uncertain areas were confirmed by OCT scans to clarify the area occupied by cuticular drusen.

All measurements were assessed by two independent retinal specialists (S.W.N. and M.K.), and in cases of disagreement, a senior investigator (D-I.H.) made the final decision.

### Statistical analysis

Quantitative variables are presented as mean ± standard deviation. Frequencies were compared between groups using the chi-square test. Analyses of continuous variables were performed using independent t-test and one-way ANOVA test. We analyzed linear correlations with Pearson’s correlation coefficient (r) for normally distributed continuous variables. Statistical significance was defined as a p-value of < 0.05. All statistical analyses were performed using SPSS software (version 20.0; SPSS, Chicago, IL, USA).

## Results

A total of 52 eyes from 32 patients were included in the study. The demographic and clinical features of the patients are summarized in Table 1. The mean age was 65.7 ± 7.3 years, and 17/52 (32.7%) patients were male. The BCVA was 0.07 ± 0.11 logMAR. The MRS was 22.9 ± 4.6 dB, MD was − 3.7 ± 4.3 dB, and PSD was 3.4 ± 2.7 dB.

**Table 1:** Clinical features in eyes with cuticular drusen.

	Cuticular drusen (n = 52)
Age (years)	65.7 ± 7.3 [50.0–77.0]
Male (%)	17/52 (32.7%)
BCVA (logMAR)	0.07 ± 0.11 [0.00–0.70]
**Microperimetry**	
Mean retinal sensitivity (dB)	22.9 ± 4.6 [3.6–27.7]
Mean deviation (dB)	− 3.7 ± 4.3 [− 23.4–0.6]
Pattern standard deviation (dB)	3.4 ± 2.7 [1.3–11.0]
Area occupied by drusen (total 52)	37.2 ± 14.5 [2.0–51.0]
Pupil size (mm)	4.6 ± 1.1 [2.1–6.3]
False positive rate (%)	0.2 ± 1.5% [0.0–11.0]

Data are total no. (%) or mean ± standard deviation, unless otherwise indicated.

*BCVA* Best corrected visual acuity, *logMAR* logarithm of the minimum angle of resolution, *dB* Decibel.

### Analyses of the clinical features between the types of cuticular drusen

**Figure 2:**
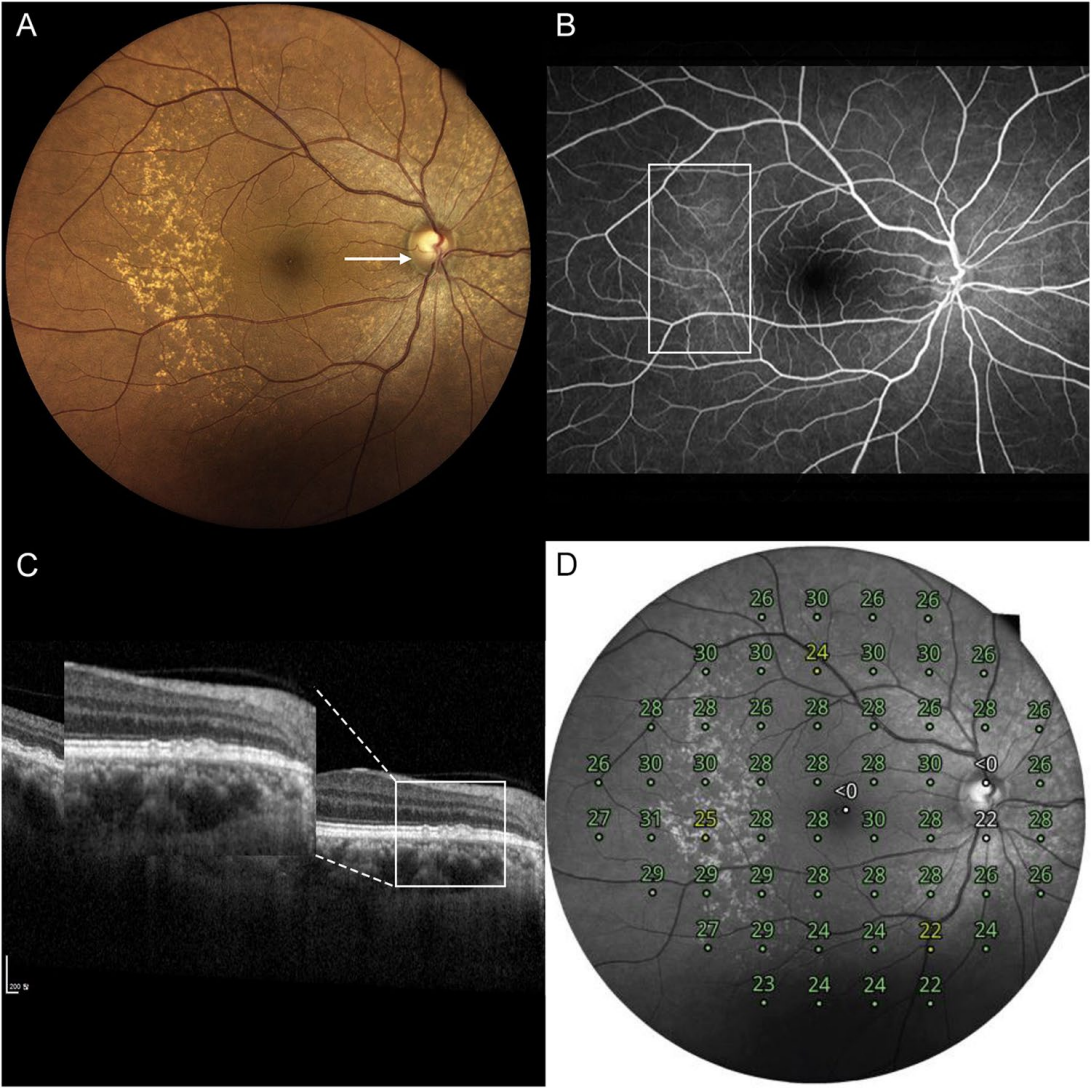
A representative case of type 1 cuticular drusen. A 57-year-old woman presented with type 1 cuticular drusen. (**A**) Color fundus photography showed numerous cuticular drusen. The cuticular drusen area was scanned using spectral-domain optical coherence tomography (SD-OCT) (arrow). (**B**) Fundus fluorescein angiography showing hyperfluorescent drusen. This can confirm the diagnosis of cuticular drusen. (**C**) In the SD-OCT image scanned on the site of the arrow in Photo (**A**), the cuticular drusen area is magnified to categorize the types of cuticular drusen. It can be diagnosed as a type 1 cuticular drusen in which shallow elevation of the retinal pigment epithelium is present in a spectralis domain optical coherence tomography image. (**D**) Retinal sensitivity using microperimetry is topographically presented in a composited image.

**Figure 3:**
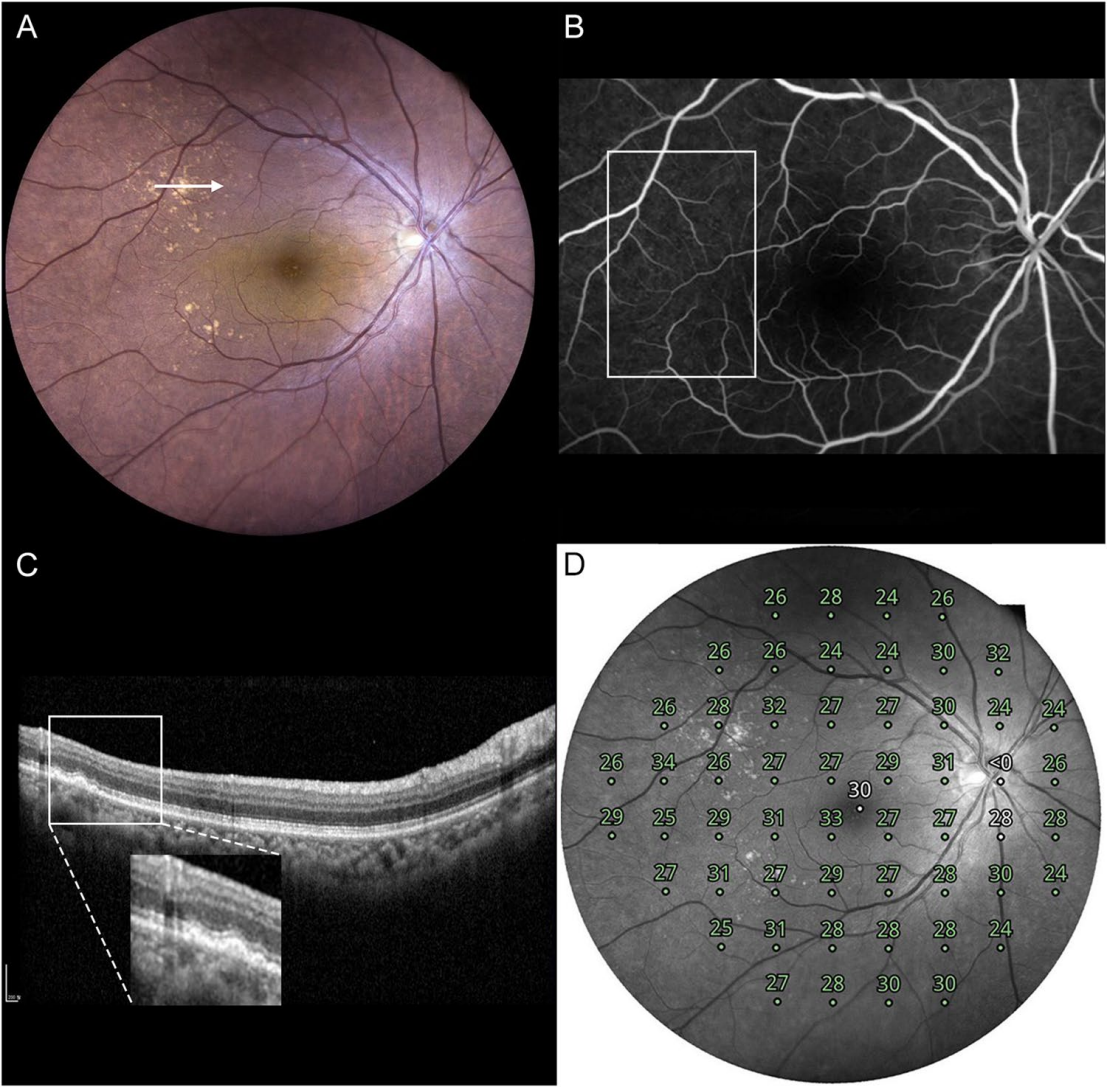
A representative case of type 2 cuticular drusen. A 70-year-old woman presented with type 2 cuticular drusen. (**A**) Color fundus photography showed numerous cuticular drusen. The cuticular drusen area was scanned using spectralis-domain optical coherence tomography (SD-OCT) (arrow). (**B**) Fundus fluorescein angiography showing hyperfluorescent drusen. This can confirm the diagnosis of cuticular drusen. (**C**) In the SD-OCT image scanned on the site of the arrow in Photo A, the cuticular drusen area is magnified to categorize the types of cuticular drusen. It can be diagnosed as a type 2 cuticular drusen in which sub retinal pigment epithelium triangular morphologic features, resulting in a saw-tooth appearance are present in a spectralis domain optical coherence tomography image. (**D**) Retinal sensitivity using microperimetry is topographically presented in a composited image.

**Figure 4:**
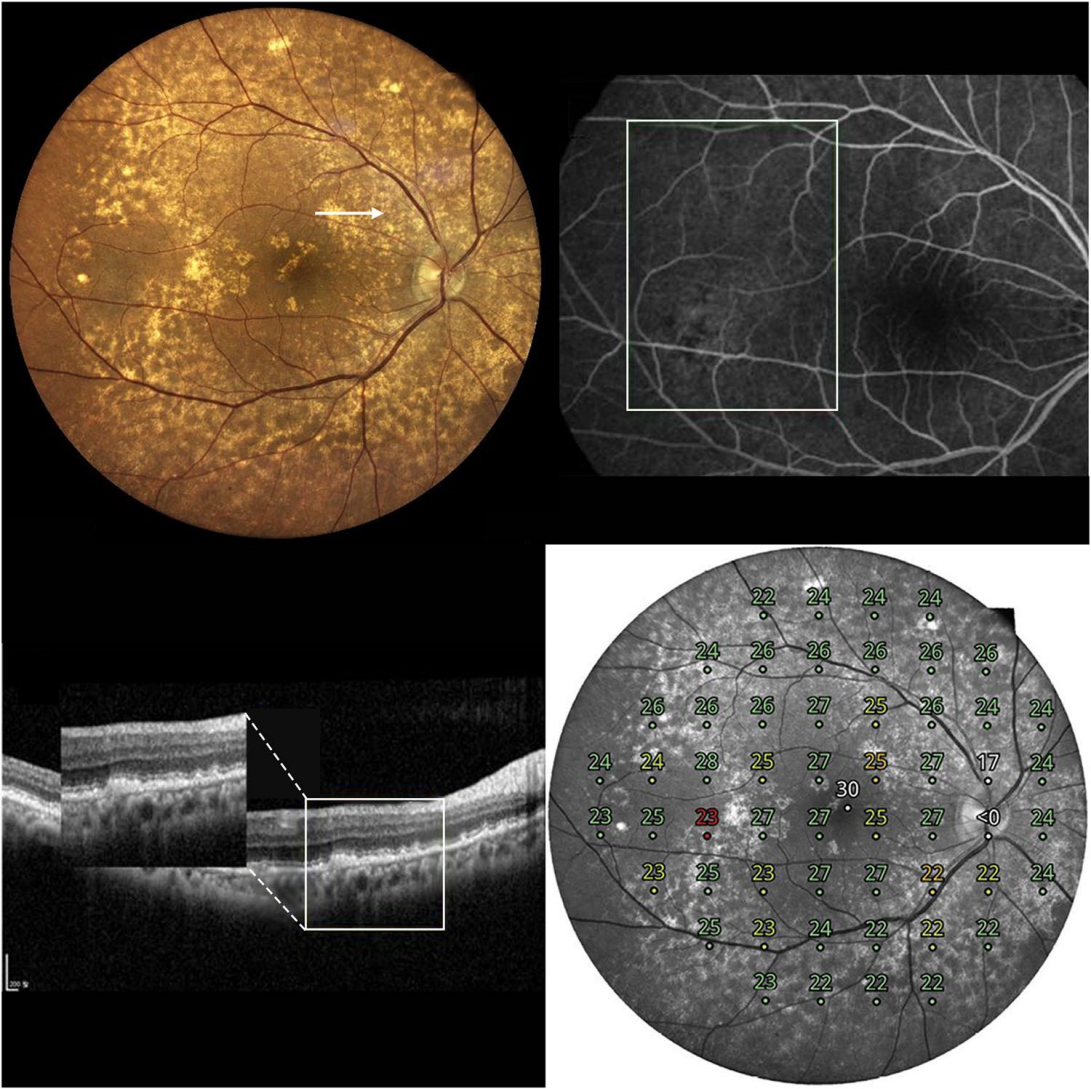
A representative case of type 3 cuticular drusen. A 66-year-old man presented with type 3 cuticular drusen. (**A**) Color fundus photography showed numerous cuticular drusen. The cuticular drusen area was scanned using spectralis-domain optical coherence tomography (SD-OCT) (arrow). (**B**) Fundus fluorescein angiography showing hyperfluorescent drusen. This can confirm the diagnosis of cuticular drusen. (**C**) In the SD-OCT image scanned on the site of the arrow in Photo (**A**), the cuticular drusen area is magnified to categorize the types of cuticular drusen. It can be diagnosed as a type 3 cuticular drusen in which broad, mound-like elevation of the retinal pigment epithyelim is present in a spectralis domain optical coherence tomography image. (**D**) Retinal sensitivity using microperimetry is topographically presented in a composited image.

Twelve (23.1%), 19 (36.5%), and 21 (40.4%) eyes had type 1, 2, and 3 cuticular drusen, respectively. Representative cases of type 1 (Fig. 2), type 2 (Fig. 3), and type 3 (Fig. 4) cuticular drusen are presented below.

The MRS of types 1, 2, and 3 was 25.1 ± 2.2 (dB), 23.4 ± 3.9 (dB), and 21.3 ± 5.7 (dB), respectively (p = 0.063, ANOVA), and the MRS of types 1 and 3 was significantly different (p = 0.011, t-test). The MD of types 1, 2, and 3 was − 1.8 ± 2.1 (dB), − 3.4 ± 3.9 (dB), and − 5.1 ± 5.3 (dB), respectively (p = 0.098, ANOVA), and the MD of types 1 and 3 was significantly different (p = 0.016, t-test). The PSD of types 1, 2, and 3 was 2.3 ± 1.0 (dB), 3.5 ± 3.0 (dB), and 4.0 ± 3.0 (dB), respectively (p = 0.219, ANOVA), and the PSD of types 1 and 3 was significantly different (p = 0.025, t-test). The factors associated with test reliability, including the pupil size and false-positive rate, did not differ between the groups (p > 0.05, ANOVA and t-test). Table 2 shows the results of the comparative analyses of the clinical features according to the cuticular drusen type.

**Table 2:** Comparative analyses of clinical features according to the types of cuticular drusen.

	Type 1 (n = 12)	Type 2 (n = 19)	Type 3 (n = 21)	*p*_*12*_	*p*_*23*_	*p*_*13*_	p_*_
Age (years)	65.6 ± 5.4	63.2 ± 8.3	68.0 ± 6.8	0.332	0.051	0.262	0.106
Male (%)	4 (33.3%)	6 (31.6%)	7 (33.3%)	0.919	0.906	1.000	0.992
BCVA (logMAR)	0.04 ± 0.05	0.08 ± 0.16	0.07 ± 0.07	0.326	0.746	0.205	0.612
**Microperimetry**							
Mean retinal sensitivity (dB)	25.1 ± 2.2	23.4 ± 3.9	21.3 ± 5.7	0.147	0.173	0.011	0.063
Mean deviation (dB)	− 1.8 ± 2.1	− 3.4 ± 3.9	− 5.1 ± 5.3	0.133	0.258	0.016	0.098
Pattern standard deviation (dB)	2.3 ± 1.0	3.5 ± 3.0	4.0 ± 3.0	0.115	0.593	0.025	0.219
Area occupied by drusen (total 52)	40.2 ± 9.2	40.5 ± 14.5	31.0 ± 16.6	0.949	0.195	0.194	0.301
Pupil size (mm)	5.0 ± 1.1	4.8 ± 1.1	4.2 ± 1.1	0.657	0.099	0.075	0.110
False positive rate (%)	0.0 ± 0.0	0.6 ± 2.5	0.0 ± 0.0	0.331	0.331	NA	0.428

Data are total no. (%) or mean ± standard deviation, unless otherwise indicated.

*BCVA* Best corrected visual acuity, *logMAR* logarithm of the minimum angle of resolution, *dB* Decibel.

*p*_*12*_: p value of comparison between type 1 and type 2.

*p*_*23*_: p value of comparison between type 2 and type 3.

*p*_*13*_: p value of comparison between type 1 and type 3.

p*: p value of comparison between type 1, type 2, and type 3.

### Analyses of the extent of cuticular drusen between the cuticular drusen type

The number of areas occupied by drusen was 37.2 ± 14.5 areas out of 52 areas. The number of areas occupied by drusen of types 1, 2, and 3 was 40.2 ± 9.2, 40.5 ± 14.5, and 31.0 ± 16.6, respectively. The areas occupied by drusen were not significantly different among the cuticular drusen types (p = 0.301, ANOVA).

### Association between retinal sensitivity and other factors

MRS was significantly related to age (r = − 0.395; p = 0.004, Pearson correlation), BCVA (logMAR) (r = − 0.315; p = 0.023, Pearson correlation), and pupil size (r = 0.375; p = 0.006, Pearson correlation).

## Discussion

In this study, retinal sensitivity differed according to the cuticular drusen type based on OCT. Type 3 cuticular drusen showed a significant reduction in retinal sensitivity, without a significant difference in the extent of cuticular drusen.

Drusen and drusenoid deposits are associated with retinal sensitivity. Soft drusen are associated with the deterioration of retinal sensitivity much earlier than visual acuity changes^11^. RPD, which is mainly characterized by the atrophy of the photoreceptor layer, can also develop deterioration of retinal sensitivity and electrophysiological retinal function, and it was related to the extent of RPD^16,17^. However, retinal sensitivity of cuticular drusen was scarce because of the low prevalence of cuticular drusen. Pfau et al.^18^ and Goh et al.^5^ reported studies of microperimetry in eyes with cuticular drusen^5^. However, in addition to cuticular drusen, they included cases with soft drusen, which can affect retinal sensitivity^5^. Charng et al. included patients with cuticular drusen only, and they compared microperimetry data between eyes with cuticular drusen and healthy controls^19^. However, they used microperimetry within 6° of the fovea despite the wide distribution of cuticular drusen on the fundus. Therefore, microperimetry data with a wide field (30°) of cuticular drusen is valuable for understanding the pathogenesis of cuticular drusen.

The composition of drusen differs between the types of drusen or drusenoid deposits^20,21^. Therefore, there may be different biological mechanisms of photoreceptor damage according to the type of drusen or drusenoid deposits. The topographic relationship of soft drusen to cones and RPD to rods strongly suggests that there are different mechanisms of progression according to the type of drusen or drusenoid deposit^20^. Cuticular drusen may have a unique mechanism of photoreceptor damage in that cuticular drusen is a unique risk factor for progression to advanced AMD^4,5^. Charng et al. reported that eyes with cuticular drusen demonstrated relative scotoma, but mean retinal sensitivity was not affected^19^. However, the age of the cuticular drusen group was young (48.5 years), they used relatively narrow field microperimetry (6° from the fovea), and they did not classify the types of cuticular drusen. In this study, because there was no age-matched control group, the effect of cuticular drusen on retinal sensitivity alone could not be revealed. Nevertheless, we investigated the differences between the types of cuticular drusen.

The types of cuticular drusen were first reported by Balaratnasingam et al.^4^ who reported that types 1, 2, and 3 cuticular drusen were found in 33%, 49%, and 18% of the cases, respectively. In this study, types 1, 2, and 3 cuticular drusen were found in 23.1%, 36.5%, and 40.4% of patients, respectively, which showed a relatively large proportion of type 3 cuticular drusen. This could be due to the older age (65.7 years vs. 57.9 years) of patients in this study. Further studies are needed to investigate the proportion of cuticular drusen and the relationship between age and cuticular drusen type.

Visual acuity is the good parameter of eye function, but is insufficiently sensitive to detect the early or late stage of macular functional loss exactly^22^. However, retinal sensitivity is effective to detect reductions of subtle retinal function^22^. Since cuticular drusen often maintains visual acuity as it progresses, we used retinal sensitivity for analysis. In this study, type 3 cuticular drusen showed marked reductions in MRS, MD, and PSD (p < 0.05), although age, the number of areas occupied by drusen, pupil size, and the false-positive rate were not significantly different among the cuticular drusen type (p > 0.05). Furthermore, the one-way ANOVA of MRS and MD among the drusen types was marginally significant (p < 0.10). Type 3 cuticular drusen can have more drusen volume than types 1 and 2 cuticular drusen. Histopathologically, retinal cells on drusen show structural and molecular abnormalities including photoreceptor degeneration and Muller glial activation, and the function of photoreceptor cell may be compromised due to drusen^23^. Zeifel et al. reported outer segment shortening and loss with inner segment deflection of photoreceptor over subretinal drusenoid deposit formation^7^. Iwama reported that drusen showed focal areas with reduced retinal sensitivity, areas that were consistent with RPE and ellipsoid zone abnormalities^24^. As with previous reports, our results may imply that elevated drusen can deform the RPE shape, and it is related to decreased retinal sensitivity. Determining the cuticular drusen type on OCT is valuable because it can functionally affect the retinal sensitivity. Additionally, microperimetry may be an important method for evaluating retinal function in cuticular drusen eyes. A larger sample size study is needed to determine a definite difference.

MRS was significantly related to age and pupil size (p < 0.05) in this study. Old age and small pupil size are well-known factors of low retinal sensitivity^25^. Therefore, this study showed similar trends to previous studies in the microperimetry results.

This study is the first to present a wide-field microperimetric analysis of cuticular drusen eyes. There have been no studies on the differences in retinal sensitivity among the types of cuticular drusen. Our study suggests that determining the cuticular drusen type on OCT is valuable, and deterioration of retinal sensitivity can develop without deterioration of vision according to the cuticular drusen type.

This study has several limitations. First, this was a retrospective cross-sectional study. Therefore, we did not observe longitudinal changes in cuticular drusen. Longitudinal studies with topographical analyses could help draw more definitive conclusions on the changes in drusen volume and retinal sensitivity in cuticular drusen eyes. Second, our study did not include age-matched healthy controls. Third, this study had a small sample size due to the low prevalence of cuticular drusen. Further studies with larger sample sizes are required.

In conclusion, this study showed that retinal sensitivity varies according to the cuticular drusen type based on OCT. Type 3 cuticular drusen showed a significant reduction in retinal sensitivity compared with type 1 cuticular drusen.

## Data Availability

The datasets generated during and/or analyzed during the current study are available from the corresponding author on reasonable request.
